# The effect of the horizontal metallic drive on reference dosimetry in the SNC 3D scanner water tank

**DOI:** 10.1002/acm2.12858

**Published:** 2020-04-01

**Authors:** Sadia Aftab, Michael P. Barnes, Marcus Doebrich, Joerg Lehmann

**Affiliations:** ^1^ Department of Radiation Oncology Calvary Mater Hospital Newcastle NSW 2298 Australia; ^2^ Institute of Medical Physics The University of Sydney Sydney NSW Australia; ^3^ Andrew Love Cancer Centre University Hospital Geelong Barwon Health Geelong Vic. Australia; ^4^ School of Medical Radiation Sciences University of Newcastle Newcastle NSW Australia; ^5^ School of Mathematical and Physical Sciences University of Newcastle Newcastle NSW Australia

**Keywords:** 3D scanner, backscatter, reference dosimetry, water tank

## Abstract

Accurate quantification of absorbed radiation dose is important for safe and effective delivery of radiation therapy. An important aspect to this is reference dosimetry, which is performed under reference conditions specified by international codes of practice. Such measurements are usually performed in a water phantom. In the Sun Nuclear Corporation (SNC) three‐dimensional (3D) scanner water tank system the detector holder is fixed to a horizontal metallic drive rail (MDR) which is in close proximity to the active volume of the detector. In this project, the dosimetric effects of the MDR on reference dosimetry were investigated for MV photons, MeV electrons, and kV photons by comparing reference dosimetry measurements in the SNC 3D scanner against similar measurements in a Standard Imaging (SI) one‐dimensional (1D) tank and against measurements in the SNC 3D scanner with an additional, custom‐made spacer placed beneath the chamber holder to increase the chamber ‐ MDR separation. A second experiment investigated the difference in chamber reading dependent on chamber to MDR separation by fixing the chamber in the tank independently of the MDR and successively moving the MDR vertically to alter the separation. The results showed that measurements in the SNC 3D scanner agree with both SI 1D tank and SNC 3D scanner with spacer to within ±0.3% for MV photons, ±0.1% for electrons and ±1.2% for kV photons within the calculated setup uncertainty. The second experiment showed that the contribution of backscatter from the MDR was significant if the distance between MDR and chamber was reduced below the chamber's designed position in the SNC 3D scanner. The exception was for kV photons where the contribution of backscatter from the MDR was measured to be 0.5% at the designed distance. Further investigation would be useful for kV photons, where the experiment showed relatively large measurement uncertainties.

## 1 INTRODUCTION

Reference dosimetry is the direct measurement of absorbed dose traceable to a standards laboratory. It is performed in radiotherapy clinics to calibrate the treatment machine Monitor Units (MU) to absorbed dose to water under reference conditions. Reference dosimetry is performed according to a recognized protocol. Examples of such protocols include IAEA TRS‐398,^1^ AAPM TG‐51,^2^ and AAPM TG‐61.^3^ These protocols specify the reference conditions at which measurements should be taken including field size, source to surface distance (SSD) and depth. The SSD and depth of calibration may vary with radiation type and beam quality but there is often a common condition of measurement in water. Measurements according to in‐water based formalisms are often performed in a scanning water tank phantom.

**FIG. 1:**
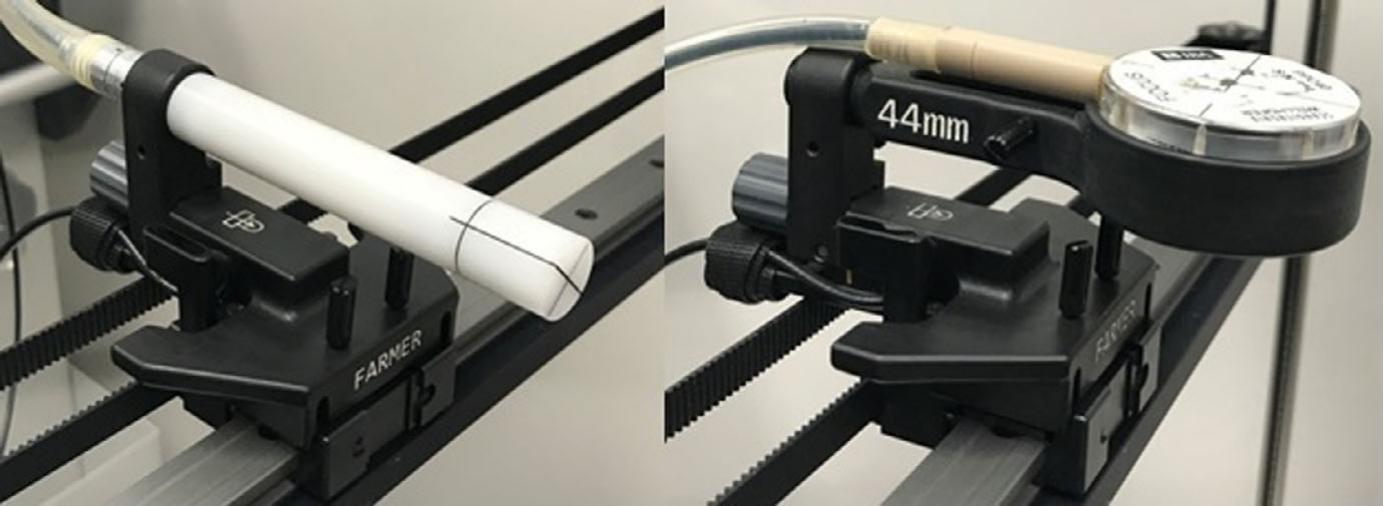
Farmer‐type and ROOS chambers installed in the Sun Nuclear Corporation (SNC) three‐dimensional scanner using the SNC provided chamber holders, shows the close proximity of chamber to metallic drive rail.

In recent years there has been significant development of scanning water tank systems. In 2015 Sun Nuclear Corporation (SNC) (Sun Nuclear Corporation, Melbourne, FL, USA) released the three‐dimensional (3D) scanner system. In this tank the detector holder is attached to a horizontal metallic drive rail (MDR) made of Aluminum, which places the detector in close proximity to the MDR (Fig. 1). The chamber's geometric point of measurement is placed 1.6 cm from the edge of the chamber holder and approximately 6 cm above the MDR for a Farmer‐type chamber and 7.65 cm for a ROOS chamber. This places the MDR downstream, but still within the field for both chamber types. The presence of the MDR in the water phantom is a deviation from standard dosimetry protocol guidelines^1^, ^2^ which stipulate for measurements to be taken in a homogeneous water phantom. In the case of megavoltage (MV) photon beams backscatter radiation from the MDR may impact the accuracy of reference dosimetry as a significant contribution of radiation backscattered from high atomic number (*Z*) materials can be expected.^4^ In megavoltage (MeV) electron beams the electrons incur large angular deflections which result in backscatter and this is most noticeable when low energy electron beams (6 MeV) strike a high‐*Z* scatterer.^5^ A reduction in dose due to loss of backscatter with kilovoltage (kV) photons has been reported by Healy et al.^6^ and found that the surface dose reduced by 2% due to the presence of lead behind the backscatter material (solid water).

The performance of the SNC 3D scanner has been investigated previously by other groups,^7^, ^8^, ^9^ but a backscatter effect of the MDR on reference dosimetry was not considered. This study investigated and characterized the impact of the MDR on reference dosimetry for MV photon, MeV electron, and kV photon beams. A series of measurements were performed to compare the dosimetric effects of the MDR on reference dosimetry in different tanks with different MDR configurations and the associated uncertainties were estimated. The backscatter effect of the MDR was also investigated by changing the separation between the MDR and the respective chamber.

## 2 MATERIALS AND METHODS

### 2.A Materials

#### 2.A.1 Water scanning systems

**FIG. 2:**
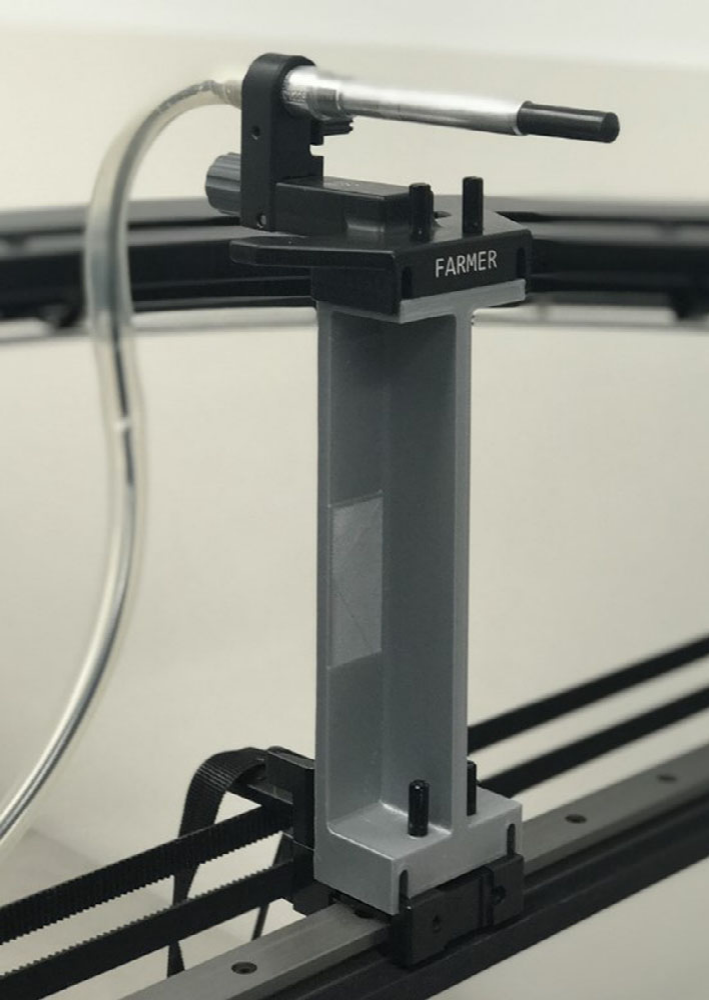
Three‐dimensional printed 14.7 cm long spacer, used in experiment 1, to increase the chamber — metallic drive rail separation.

The charge collected by an ion chamber was measured under reference conditions in two different water tanks, the SNC 3D water scanning system and a Standard Imaging (SI) (Standard Imaging, Inc. Middleton, WI, USA) one‐dimensional (1D) water tank, which does not have a MDR. In the SI 1D tank, the chamber attaches to a 1D scanning arm via a holder. The scanning arm can only move vertically and is attached to the wall of the phantom and hence there is no horizontal drive rail in the beam. Measurements were performed in the SNC 3D system with the inclusion of a custom spacer (approximately 14.7 cm in length, Fig. 2) placed between the chamber holder and the MDR to increase the separation. The spacer was printed from Polylactic Acid (PLA) material using an Ultimaker 3 Extended 3D printer (Ultimaker B.V., Geldermalsen, The Netherlands).

#### 2.A.2 Treatment units

This study was carried out on a Varian Clinac iX (Varian Medical System, Palo Alto, CA, USA) using two beam modalities: photons with nominal energies of 6 MV and 18 MV and electrons with energies of 6, 12, and 20 MeV. The WOmed T‐200 (WOmed WOLF‐Medizintechnik GmbH, An Eckert & Ziegler BEBIG Company, Germany) therapy system was used to deliver kV photons of 100 and 200 kV.

#### 2.A.3 Dosimeters

A 0.6 cc cylindrical Farmer‐type ionization chamber (FC65‐G, IBA Scanditronix Wellhöfer, USA) was used for all photon measurements and a plane parallel chamber (ROOS, IBA Scanditronix Wellhöfer, USA) was used for the electron beams. For measurements in MV beams each chamber was connected to a Farmer electrometer (NE2570/1, Thermo Nuclear Enterprises, UK) as per departmental reference dosimetry procedures. In the kV photon beams the FC65‐G chamber was used with a Webline Unidos electrometer (PTW Freiburg, Germany) as per departmental protocol. All measurements were performed with –300 V bias voltage applied.

### 2.B Methods

The impact of the MDR on reference dosimetry measurements was investigated in two experiments:

1. Comparison of reference dosimetry in different tanks/setups
2. Assessment of the impact of backscattered radiation on the measured signal as a function of the distance between the chamber and the MDR

For all MV measurements the linac gantry's true zero was found using a precision spirit level prior to levelling the water tank. The source to surface distance (SSD) was set to 100 cm using a reference front pointer and the depth was set using the tank drive mechanism which was verified using a steel ruler. The reference field size was set to 10 × 10 cm^2^ for MV photons and the 15 × 15 cm^2^ reference applicator was used for MeV electrons. For the kV measurements the reference applicator (8 × 10 cm^2^) was attached to the unit head and levelled using the spirit level. The reference applicator has a fixed FSD of 40 cm. The bottom of the applicator was positioned at the water surface. The central axis was placed precisely over the chamber's sensitive volume by using a setup cap for guidance.

The respective electrometer was always switched on and the bias voltage was applied at least 15 min prior to the measurements. A digital thermometer (Reference Thermometer, Electronic Temperature Instruments Ltd, UK) was mounted inside the water tank to record the water temperature and a precision barometer (HHP360 – B, Omega Engineering, USA) was used to record air pressure. For warm‐up, to stabilize its response, the ion chamber was exposed to approximately 5 Gy at the beginning of each measurement session before taking readings.

#### 2.B.1 Experiment 1: Comparison of reference dosimetry in different water tanks

In experiment 1, the reference dosimetry measurements were compared in different water tanks to quantify the effect of the MDR on reference dosimetry results. Three setup configurations were used to investigate the scatter contribution from the MDR:

1. Measurements in the SNC 3D scanner (setup as recommended by the manufacturer)
2. Measurements in the SNC 3D scanner with a 14.7 cm spacer placed between the chamber and the MDR
3. Measurements in the SI 1D water tank which does not have a MDR.

The measurements were made along the central axis under reference conditions as per the IAEA TRS‐398 reference dosimetry protocol^1^ with the chamber at the respective reference depth for MV photon and MeV electron beams and at 2 cm reference depth as recommended in the AAPM TG‐61^3^ dosimetry protocol for kV beams. A total of three measurement sessions were performed for each radiation type; the tank was set up separately for each session. 200 MU were delivered for each measurement at a dose rate of 600 MU/min for MV photons and MeV electrons and 120 s exposure time was used for the kV photon beams.

Each time the tank was set up there were associated setup uncertainties. The same beam energies, chamber, and electrometer combinations were used in each setup. Hence, all reference dosimetry perturbation and calibration factors were common and therefore cancelled out in comparison. Polarity and ion recombination were measured once with each tank to determine whether the MDR had an effect on these quantities. The variations in linac jaw positions as well as gantry and collimator angles between tank setups were considered insignificant based on monthly quality assurance results. Measurements were corrected for temperature and pressure. Therefore, uncertainties between tank setups were dominated by uncertainties in setting the SSD and the chamber depth. The dosimetric effect of the SSD‐related setup uncertainty was modelled using the inverse square law with the departmental virtual source position used for electron beams and an assumed maximum uncertainty of ±1 mm in the front pointer measurement. Percentage depth dose (PDD) curves were used to estimate the dosimetric effect of the uncertainty in setting the chamber depth assuming a 1 mm error in depth setting. The total setup uncertainty was calculated by adding the SSD and depth dosimetric uncertainties in quadrature. Additionally, the reproducibility was calculated as one standard deviation (SD) across the three measurement sessions.

#### 2.B.2 Experiment 2: Impact of backscattered radiation as a function of the chamber ‐ MDR distance

The impact of backscatter from the MDR as a function of the chamber ‐ MDR separation was investigated for MV and kV photons and MeV electrons in two measurement sessions, each under reference conditions following the TRS‐398 protocol^1^ for MV photons and electrons and AAPM TG‐61^3^ guidelines for kV photons.

**FIG. 3:**
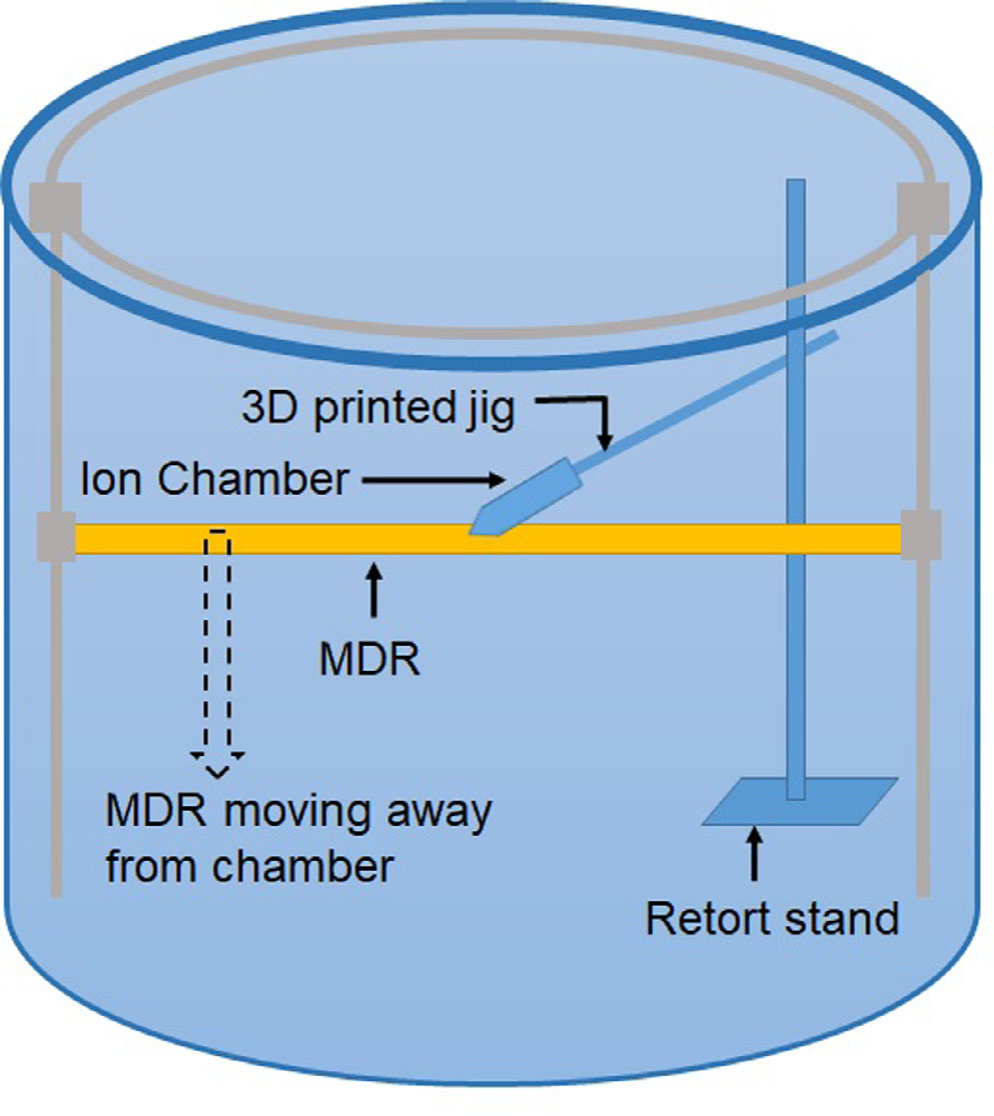
Experiment 2 setup arrangement. Ion chamber placed at reference depth in the Sun Nuclear Corporation three‐dimensional (3D) scanner using a retort stand and a 3D printed holder. The metallic drive rail (MDR) was moved independently of the camber to vary the chamber — MDR separation.

A 3D printed chamber holder was attached to a retort stand which was then placed in the SNC 3D scanner well outside the primary beam to position the chamber at the reference depth independent of the tank drive mechanism (Fig. 3). A levelling laser (Multi‐Liner FL55 Plus, geo‐FENNEL, Germany) was used to verify the reference depth in the water tank. The levelling laser was placed on the patient couch to match the water surface and then the couch was lowered to position the laser at the respective reference depth. The reference depths were also verified using a steel ruler. While the chamber remained fixed at the reference depth, the MDR was moved to different vertical distances from the chamber. Chamber charge readings were recorded at each distance. The temperature and pressure corrected readings were then normalized to the readings measured at the tanks designed chamber — MDR separation (approximately 6 cm in case of the Farmer‐type chamber and 7.65 cm for the ROOS chamber).

## 3 RESULTS AND DISCUSSION

### 3.A Experiment 1: Comparison of reference dosimetry in different water tanks

#### 3.A.1 Polarity and ion recombination

The polarity effect was measured in all tank setups and the maximum difference was found to be 0.07% for MV photons and 0.01% for MeV electrons. The measured ion recombination agreed within 0.04% for MV photons and 0.00% for MeV electrons in both tanks.

#### 3.A.2 Measurement uncertainty

The measurement uncertainties related to setting the SSD and depth (Table 1) were larger for kV photons compared to the megavoltage beams because of the short SSD and the higher gradient of the PDD curve at the reference depth. For 6 MV, 1 mm variation in position yielded an uncertainty of about 0.5% while for 100 kV 1 mm change in position resulted in an uncertainty of 2.13% in dose.

**TABLE 1:** Setup uncertainties for all energies. Variation in source to surface distance (SSD) modelled using inverse square law and variation in depth modeled using percentage depth dose.

	6 MV	18 MV	6 MeV	12 MeV	20 MeV	100 kV	200 kV
SNC 3D scanner							
SSD_InvSq_ (%)	0.20	0.20	0.20	0.20	0.20	0.50	0.50
Depth_PDD_ (%)	0.46	0.13	0.10	0.26	0.35	2.10	0.60
Total Uncer. (%)	0.50	0.24	0.22	0.29	0.40	2.13	0.74
SNC 3D scanner with spacer							
SSD_InvSq_ (%)	0.20	0.20	0.20	0.20	0.20	0.50	0.50
Depth_PDD_ (%)	0.46	0.13	0.10	0.21	0.35	2.10	0.60
Total Uncer. (%)	0.50	0.24	0.22	0.29	0.40	2.13	0.74
SI 1D tank							
SSD_InvSq_ (%)	0.20	0.20	0.20	0.20	0.20	0.50	0.50
Depth_PDD_ (%)	0.58	0.13	0.10	0.21	0.35	2.70	1.40
Total Uncer. (%)	0.61	0.24	0.22	0.29	0.40	2.78	1.53
Combined uncertainties added in quadrature (%)							
SNC 3D scanner vs SNC 3D scanner with spacer	0.71	0.34	0.31	0.41	0.57	3.01	1.05
SNC 3D scanner vs 1D tank	0.79	0.34	0.31	0.41	0.57	3.50	1.70

1D, one‐dimensional; 3D, Three‐dimensional; SNC, Sun Nuclear Corporation.

#### 3.A.3 Comparison of tanks

The measurements for experiment 1 (Table 2) agreed among tank setups within the uncertainty for all energies. For MV photons, a maximum difference of 0.3% was measured with a calculated setup uncertainty of 0.79%, and for MeV electrons, a maximum difference of 0.1% was measured with a calculated setup uncertainty of 0.57%. For 100 kV photons a maximum difference of −1.2% was measured with a calculated setup uncertainty of 3.01%. The reproducibility between the three independent measurement sessions (0.64%) was within the setup uncertainties which may indicate that the uncertainty assumption of 1 mm in both SSD and depth may have been too conservative. A maximum difference of 1.1% was measured for 200 kV photons with a calculated setup uncertainty of 1.70% and a reproducibility of 0.37% (Table 2). This difference was in the opposite direction to the SNC 3D scanner with and without spacer setup which indicated that the results from the SNC 3D scanner with spacer did not agree with the SI 1D tank. The uncertainties of this experiment were generally large in the clinical context which meant that definitive conclusions about the clinical significance of the MDR on reference dosimetry could not be made from this experiment in isolation for kV beams.

**TABLE 2:** Reference dosimetry difference (%) in two different tanks with reproducibility (%) and associated setup uncertainty (%).

Beam	SNC 3D scanner vs SNC 3D scanner with spacer			SNC 3D scanner vs 1D tank		
	Mean difference (%)	Reproducibility (1 SD) (%)	Setup uncertainty (%)	Mean difference (%)	Reproducibility (1 SD) (%)	Setup uncertainty (%)
6 MV	−0.1	0.07	0.71	0.3	0.06	0.79
18 MV	0.0	0.03	0.34	0.2	0.03	0.34
6 MeV	0.0	0.07	0.31	0.1	0.07	0.31
12 MeV	0.0	0.14	0.41	0.0	0.21	0.41
20 MeV	0.1	0.06	0.57	−0.1	0.25	0.57
100 kV	−1.2	0.64	3.01	0.0	0.37	3.50
200 kV	−0.7	0.43	1.05	1.1	0.32	1.70

1D, one‐dimensional; 3D, Three‐dimensional; SNC, Sun Nuclear Corporation.

### 3.B Experiment 2: Impact of backscattered radiation as a function of the chamber ‐ MDR distance

**FIG. 4:**
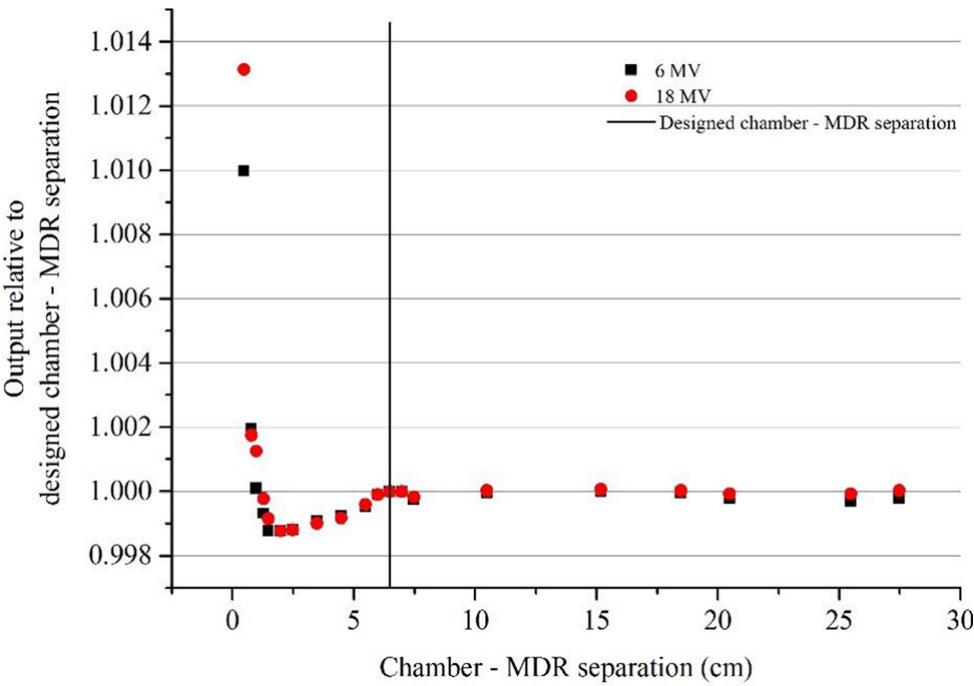
Relative output against separation between the effective point of measurement of the chamber and the metallic drive rail (MDR) for MV photons. The vertical mark represents the designed chamber — MDR separation in the three‐dimensional scanner for the Farmer‐type chamber which is the distance when the chamber is placed in the Sun Nuclear Corporation supplied standard holder mounted on the MDR.

**FIG. 5:**
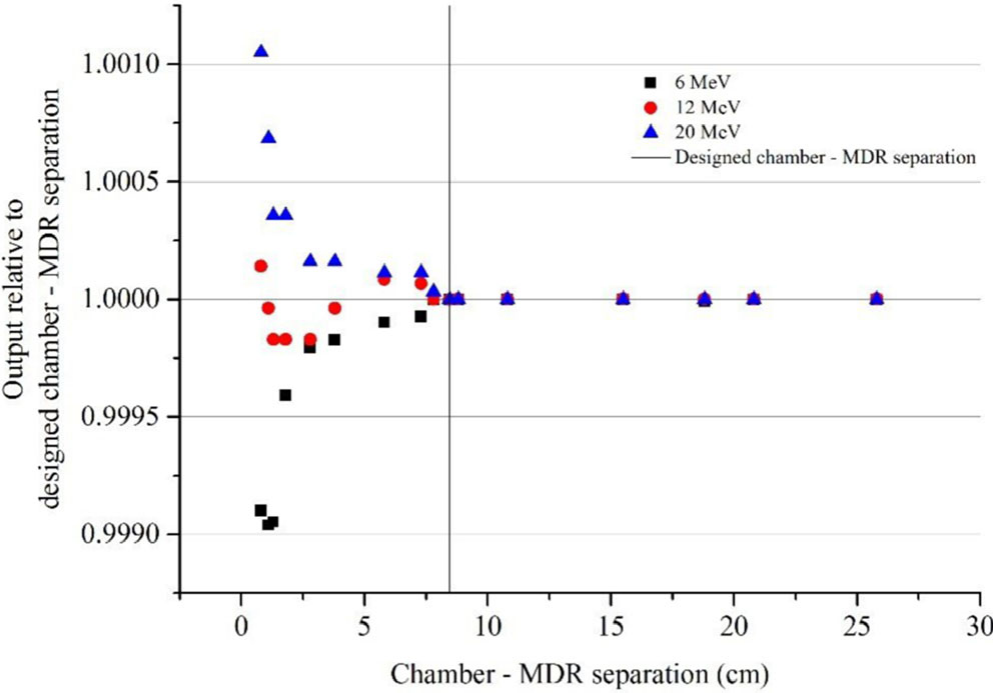
Relative output against separation between the effective point of measurement of the chamber and the metallic drive rail (MDR) for MV electrons. The vertical mark represents the designed chamber — MDR separation in the three‐dimensional scanner for the ROOS chamber which is the distance when the chamber is placed in the Sun Nuclear Corporation supplied standard holder mounted on the MDR.

**FIG. 6:**
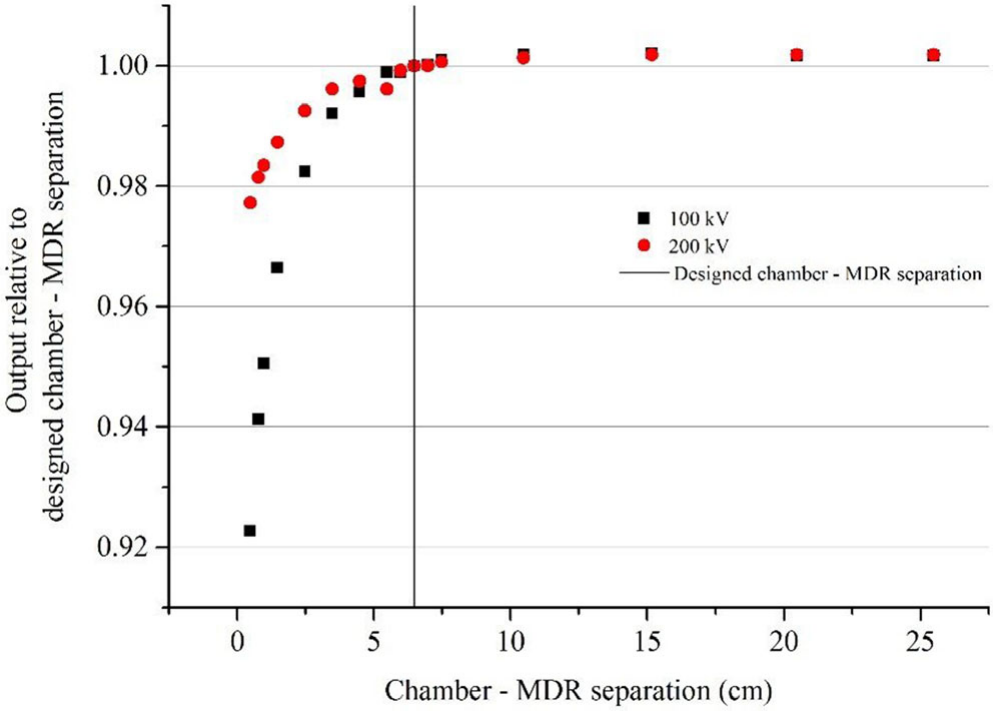
Relative output against separation between the effective point of measurement of the chamber and the metallic drive rail (MDR) for kV photons. The vertical mark represents the designed chamber — MDR separation in the three‐dimensional scanner for the Farmer‐type chamber which is the distance when the chamber is placed in the Sun Nuclear Corporation supplied standard holder mounted on the MDR.

For all beams, reducing the chamber ‐ MDR separation resulted in a measurable change in chamber readings (Figs. 4, 5, 6).

For both megavoltage photons and electrons, at the designed chamber — MDR distance, there was no significant change in readings compared to a large distance (25 cm) at which the influence of the MDR was expected to be minimal (Figs. 4 and 5).

For MV photons the measured signal at short chamber — MDR separation increased (Fig. 4). It is hypothesized that this is due to an increase in Compton backscatter from the MDR at this energy. The intensity of the backscattered radiation decreases as the distance between chamber and MDR increases. An increase in measured dose at the interface of high‐Z materials was also reported by Das et al.^10^ The range of the backscattered electrons is limited to a few millimeters.

For electron beams (Fig. 5) the phenomenon of loss of backscatter was evident for the 6 MeV electron beam. Klevenhagen^11^, ^12^ studied the electron backscatter behavior and found that the electron backscatter factor decreases with increasing energy and the exponential reduction in penetration of backscatter observed with depth. When the electron beam strikes a high‐*Z* scatterer, the effective electron energy drops off with depth and the resultant scattered electrons have a small range, which increases with increased electron energy.^11^, ^12^, ^13^ However, the magnitude of the effect shown in the scale of Fig. 5 is very small which makes it difficult to draw a definitive conclusion.

For kV photons, the measured signal decreased for small chamber — MDR separations (Fig. 6). At these separations (from 0 to 6 cm) the reduction in the chamber reading was potentially due to a loss in backscatter because of increased photoelectric absorption by the MDR in this energy range. This effect increases with decreasing photon energy in the kV range,^14^ which explains why the effect is greater for 100 kV than for 200 kV. At separation distances greater than the designed distance (>6 cm) the relative output increased to approximately 0.5% at 25 cm distance, where the effect of the MDR can be considered minimized. This indicated that at the designed separation the MDR had an influence on the measurements.

The results of experiment 2 showed that a measurable amount of backscattered radiation from the MDR existed at the short chamber — MDR separation. But beyond the designed distance in the SNC 3D scanner, the MDR effect was minimal except for kV photons.

## 4 CONCLUSION

At the designed distance between the chamber and the MDR, the impact of the MDR on reference dosimetry appeared clinically insignificant except for kV photons. Further investigation is indicated for kV photons since the experiment showed relatively large measurement uncertainties. This could potentially be investigated using Monte Carlo simulations. An option could be to include the effect of the MDR in the uncertainty budget for TG‐61 measurements when using this tank.

## CONFLICT OF INTEREST

Calvary Mater Newcastle is a reference site for the SNC 3D scanner water tank.
